# Using Convolutional Neural Networks to Derive Neighborhood Built Environments from Google Street View Images and Examine Their Associations with Health Outcomes

**DOI:** 10.3390/ijerph191912095

**Published:** 2022-09-24

**Authors:** Xiaohe Yue, Anne Antonietti, Mitra Alirezaei, Tolga Tasdizen, Dapeng Li, Leah Nguyen, Heran Mane, Abby Sun, Ming Hu, Ross T. Whitaker, Quynh C. Nguyen

**Affiliations:** 1 Department of Epidemiology and Biostatistics, University of Maryland School of Public Health, College Park, MD 20742, USA; 2 Walt Whitman High School, Bethesda, MD 20817, USA; 3 Department of Electrical and Computer Engineering, Scientific Computing and Imaging Institute, University of Utah, Salt Lake City, UT 84112, USA; 4 Department of Geography and Geospatial Sciences, South Dakota State University, Brookings, SD 57007, USA; 5 Department of Health Policy and Management, University of Maryland School, College Park, MD 20742, USA; 6 Public Health Science Program, University of Maryland School, College Park, MD 20742, USA; 7 School of Architecture, Planning & Preservation, University of Maryland School, College Park, MD 20742, USA; 8 School of Computing, Scientific Computing and Imaging Institute, University of Utah, Salt Lake City, UT 84112, USA

**Keywords:** built environment, big data, GIS, computer vision, structural determinants of health, machine learning

## Abstract

Built environment neighborhood characteristics are difficult to measure and assess on a large scale. Consequently, there is a lack of sufficient data that can help us investigate neighborhood characteristics as structural determinants of health on a national level. The objective of this study is to utilize publicly available Google Street View images as a data source for characterizing built environments and to examine the influence of built environments on chronic diseases and health behaviors in the United States. Data were collected by processing 164 million Google Street View images from November 2019 across the United States. Convolutional Neural Networks, a class of multi-layer deep neural networks, were used to extract features of the built environment. Validation analyses found accuracies of 82% or higher across neighborhood characteristics. In regression analyses controlling for census tract sociodemographics, we find that single-lane roads (an indicator of lower urban development) were linked with chronic conditions and worse mental health. Walkability and urbanicity indicators such as crosswalks, sidewalks, and two or more cars were associated with better health, including reduction in depression, obesity, high blood pressure, and high cholesterol. Street signs and streetlights were also found to be associated with decreased chronic conditions. Chain link fence (physical disorder indicator) was generally associated with poorer mental health. Living in neighborhoods with a built environment that supports social interaction and physical activity can lead to positive health outcomes. Computer vision models using manually annotated Google Street View images as a training dataset were able to accurately identify neighborhood built environment characteristics. These methods increases the feasibility, scale, and efficiency of neighborhood studies on health.

## 1. Introduction

The built environment plays a crucial role in determining community health. Neighborhood characteristics have been found to influence the development of asthma and other respiratory diseases [1], cardiovascular disease [2], diabetes [3], and overall mortality [4]. In addition to physical diseases and concerns, neighborhood characteristics can influence behavioral and psychological health. Neighborhood structures can impact the accessibility of physical activity [5] and social interaction [6], both of which are vital determinants of a plethora of behavioral and psychological health concerns, namely obesity, depression, and other isolation-based mental illnesses [6]. While an unsupportive environment can be a barrier to physical activities, a supportive built environment can promote an active lifestyle by providing more safe and walkable spaces that can encourage community members to walk, bike, jog, or run in the neighborhood [7,8]. Physically active communities have better health outcomes compared to those that lack such resources [9]. A neighborhood with the presence of shared spaces and walkable streets also supports increased social connection, while a neighborhood with dilapidated buildings and traffic noise has been shown to hinder social interaction [10]. Indeed, the promotion of social integration holds conspicuous importance for all individuals, but children and older adults are affected more drastically; children who grow up in neighborhoods with such socially limiting factors have been found to have higher rates of depression, psychological distress, and diminished social and motor skills [10], while older adults were observed to have lower perceived social support and higher rates of psychological distress [6].

Previously, neighborhood audits were typically carried out with in-person site visits to identify neighborhood characteristics [11]. While this provided extremely valuable and detailed information, it limited the scale at which neighborhood research can be conducted given the expense of staff time and travel time. Other studies utilized interviews or surveys to ask city planners, community leaders or residents to report on neighborhood characteristics but these studies were often limited to certain areas or cities. Examples include the Boston Neighborhood Survey [12] and the Project on Human Development in Chicago Neighborhoods [13]. However, neighborhood surveys that included rural areas were less common. Additionally, other neighborhood studies utilized geolocalized data such as census sociodemographics, population density, and recreational opportunities available at the neighborhood level. However, little data exist on the neighborhood built environment. Our study uses a massive publicly available data resource, Google Street View images, to address the dearth of data on national built environment characteristics. Previous research groups used this data source to conduct virtual audits through manual annotation of images. In particular, researchers from the Computer Assisted Neighborhood Visual Assessment System (CANVAS) have developed a reliable and valid methodology for virtual neighborhood audits [14]. In our study, we further advance the field by leveraging computer vision to automate the process of detecting neighborhood features of interest, which allows our study to be conducted at a much larger size than previous studies, including neighborhoods across the United States.

Rather than exploring the built environment and gathering neighborhood characteristic information on site which is expensive and time consuming, we used Google Street View imagery, an innovative and publicly available geographic data source for conducting large scale studies [8]. The possibility of utilizing national data from the real world introduces new opportunities in examining built environment characteristics [15]. Google Street View (GSV) offers international coverage of street panoramas that exhibit detectable built environment characteristics. The use of GSV removes the invasiveness and infeasibility inherent to other forms of surveying by removing the need for travel to neighborhoods, instead compiling a centralized digital data collection that can be analyzed remotely and yields a massive increase in productivity [16]. In addition, GSV’s Application Programming Interface (API) offers static views that align with parameterized Hypertext Transfer Protocol (HTTP) requests. API-acquired photos can be utilized to create indicators of specific characteristics, acting as a standardized data set capable of revealing correlations between said characteristics and chronic health risks. 

Neighborhood characteristics are difficult to assess on a large scale. As a result, there is a lack of sufficient data that can help us investigate neighborhood characteristics as structural determinants of health on a national level. This lack of data hinders the ability to direct policy to mitigate harm to public health. In some instances, such as the Centers for Disease Control and Prevention’s (CDC) Healthy Communities Program, there is a focus around environmental changes to improve people’s health [17]. However, this program is no longer funded on a national level, and there is a staggering lack of programs of the same caliber that are based on socio-ecological research. In order to stimulate further public policy and action around neighborhood conditions, an increased understanding of the built environment forces that alter health must be developed. Additionally, mapping technologies have supplied new sources of data that can inform public health research. In this study, we leveraged Google Street View data and computer vision to add to the nascent field of built environment research examining neighborhood conditions as a promising, fundamental contributing factor to community health.

### Study Aims and Hypotheses

In this study, we constructed a variety of neighborhood indicators that have been theoretically and empirically linked with health outcomes. These include markers of higher urban development (two or more cars, street lights, street signs), walkability (crosswalks, sidewalks, presence of apartment and commercial buildings), and physical disorder (chain-link fence). Single lane roads were used as a marker of lower urban development. We leverage a national database of 164 million Google Street View images and analyze the images using computer vision to produce indicators of neighborhood walkability, physical disorder, and urban development. We then utilized this data source, whose scale would not be possible without computer automation, to examine national patterns associated with key health outcomes including chronic conditions and health behaviors, which are drivers of morbidity and mortality in the United States. We hypothesized that communities that are more walkable and have higher urban development will have a lower prevalence of chronic conditions and health risk behaviors. Additionally, we hypothesized that communities with higher levels of physical disorder will have a higher prevalence of chronic conditions and higher health risk behaviors.

## 2. Materials and Methods

### 2.1. Google Street View Image Collection

#### 2.1.1. Data Collection

To arrive at a national sample of Google Street View images, we utilized U.S. road networks. Google Street View cars drove across the country and captured images, such that we can only sample points across road networks that can be traveled via car. We sampled latitude and longitude coordinates every 100 m of road and downloaded images at four angles covering 0, 90, 180 and 270 degrees from each sampled location. Using the set of sampling coordinates and Google Street View’s Static Application Programming Interface (API), we collected 164 million Google Street View images with 640 × 640 pixel resolution in November of 2019.

#### 2.1.2. Data Processing

Convolutional Neural Networks (ConvNets) are a class of multi-layer deep neural networks that have been shown to be incredibly effective in identifying and extracting features from data. They are commonly used for image classification, object recognition, and analyzing structured arrays of data [18]. ConvNets offer an efficient, more scalable, and reliable approach that transforms an input into an output through many layers that learn to detect different features of a given image. In other words, ConvNets are machine learning models that can take an input image, assign weights to various characteristics in the image, learn those characteristics, and differentiate one from the other [19]. Some of the key applications of ConvNets include facial recognition [20], and extreme weather detection [21].

To improve model performance and avoid overfitting, we tried to overcome the shortcomings of using a small dataset of labeled images by pretraining models on imageNet. ImageNet is a large database with over 14 million images that were hand-annotated with more than 22,000 categories [22]. To optimize model architecture, an ImageNet pretrained ConvNet model can be “fine-tuned” (known as applying model parameter adjustments) using a smaller amount of training data from the targeted task [23]. Model performance improvements can be delivered using this method even without requiring the extensive training data and computational resources needed to train the original ConvNet.

#### 2.1.3. Built Environment Indicators

Selection of the indicators was driven by the neighborhood literature and the desire to expand upon this literature for some understudied neighborhood characteristics. Green streets were utilized to indicate access to green space, which has been shown to be health beneficial for mental and physical health [24,25]. Single lane roads are more common in rural areas and in residential neighborhoods [26], and thus were utilized as an indicator of lower urban development. Single lane roads can limit the number of cars and hence the flow of people and thus also lower social interactions. The presence of non-single family homes can indicate a mixture of residential and commercial buildings, and thus was utilized as an indicator of mixed land use. Mixed land use has been connected with health benefits because it can allow for greater access to resources [27]. Sidewalks and crosswalks were utilized as indicators of walkability, and have been connected with increased physical activity and better health outcomes [16,28,29,30]. Visible utility wires overhead can influence residents’ view of the aesthetic appeal of their neighborhood and hence were utilized in this study as an indicator of physical disorder. There is sparse literature on this indicator, especially among U.S. studies. Abroad, visible utility wires have been linked with electrocution risk [31]. Similarly, the use of chain-linked fences is understudied in the neighborhood literature. Because chain-linked fences are more temporary than other types of fencing and are commonly used in urban environments to encompass abandoned lots [32], we utilized this as an indicator of physical disorder. 

To create a training dataset for our computer vision models, we manually annotated 18,700 images (from Chicago, Illinois; Salt Lake City, UT; Charleston, West Virginia; and a national sample) from December 2016 to February 2017. These locations were chosen to capture heterogeneity in neighborhood environments across geographically and visually distinct places with varying population densities, urban development, and demographics. Labelers included the principal investigator and three graduate research assistants. Inter-rater agreement was above 85% for all neighborhood indicators. Each image received labels for these binary neighborhood characteristics: (1) street greenness (trees and landscaping comprised at least 30% of the image), (2) presence of a crosswalk, (3) single lane road, (4) building type (single-family detached house vs. other), and (5) visible utility wires.

#### 2.1.4. Computer Vision Model Building and Validation Results

**Figure 1:**
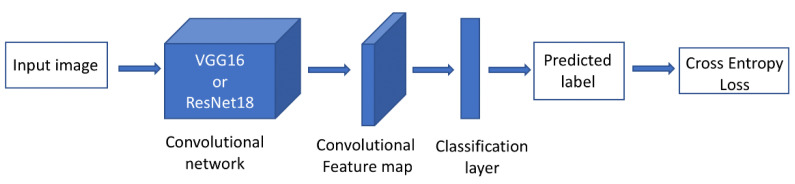
Computer vision model. Each sample is a single image accompanied by labels corresponding to each neighborhood feature (e.g., crosswalk). The feature extractor is VGG-19 or ResNet18 (depending on the feature analyzed) and is pretrained with ImageNet data. Each feature classifier is a single fully connected layer and the losses are cross entropy. The final loss for optimization is a summation of losses.

Figure 1 displays the network used for built environmental feature classification. The network is composed of two main parts, a feature extractor network that extracts built environment features from GSV images and a feature classifier network that assigns a binary label of 0 or 1 to each single image for a specific indicator. The predicted label then represents whether the corresponding indicator is presented in the image (e.g., crosswalk present or absent). For each indicator mentioned above, we used a separate classifier network, but the feature extractor part of the network was shared among all the feature classifiers. We observed that sharing the feature extractor resulted in a slight performance gain of the network as well as a reduction in training time. We used the VGG19 network and ResNet18 as our feature extractor network and a single fully connected layer as our feature classifier network. Below, we further describe the specific model building process for each group of neighborhood built environment characteristics. 

To build our computer vision model, we randomly split our manually annotated dataset. The training and validation sets contained 80% of the total labeled images, and the remaining 20% was used as a testing set to evaluate the model’s performance. Once the hyper-parameters were chosen, each model architecture was trained multiple times. Note that neural network training is stochastic even when starting from the same initialization and using the same training set. Therefore, multiple training runs were used to assess the mean and standard deviation of the error. The testing set remained unobserved until the best models were selected using the training set, and we assessed the final quality of the model using the testing set. For the training process, we first resized all the images to the size of 224 × 224. We then trained a standard deep convolutional neural network architecture, Visual Geometry Group VGG-19 [33], in TensorFlow [34] with sigmoid cross entropy with logits as the loss function. The weights of the network were initialized from ImageNet weights. Adam optimizer was used with a batch size of 20. Training took 20 epochs and started with a learning rate of 1 × 10^−4^. We considered the model saved in the last epoch as our final model. The accuracy of the recognition tasks (agreement between manually labeled images and computer vision predictions) was as follows: street greenness (88.70%), presence of crosswalks (97.20%), non-single family homes (82.35%), single lane roads (88.41%), and visible utility wires (83.00%). Below, we describe the model building process for two additional neighborhood indicators that utilize different training datasets.

To construct our sidewalks indicator, we utilized a training dataset consisting of about 24,316 images captured by Google Street View in New Jersey that had been manually labeled. We randomly split this dataset in the ratio of 80:20 for validation to obtain 19,452 images for training and 4864 for validation. The minority label images were oversampled so that the dataset has an equal number of sidewalk present and absent cases. We then trained a standard deep convolutional neural network architecture, ResNet-18 [35] in PyTorch [36], with NLL loss as the loss function. For the sidewalk indicator, the ResNet-18 model produced an accuracy of 84.5% and an F1 score of 81.0%.

To construct other urban landscape indicators, we randomly sampled 18,000 images from our national collection of GSV images. We divided this dataset into a training (80%) and testing set (20%). Quality control statistics are as follows: street lights (accuracy was 88% and F1-score was 60%); two or more cars (accuracy was 88% and F1-score was 79%); street signs (accuracy was 82% and F1-score was 68%); chain-link fence (accuracy was 95% and F1-score was 45%).

To create neighborhood summaries for each GSV-derived built environment characteristic, we utilized the latitude and longitudinal coordinates associated with each image to assign them to a census tract. Then, for each census tract, we calculated the percentage of the total number of images that contained a given built environment indicator (e.g., number of images with a sidewalk/total number of images)*100 = percent with sidewalk. From there, we created tertiles and classified each census tract based on their percentage, with the lowest tertile as the reference group.

### 2.2. Geoportal

**Figure 2:**
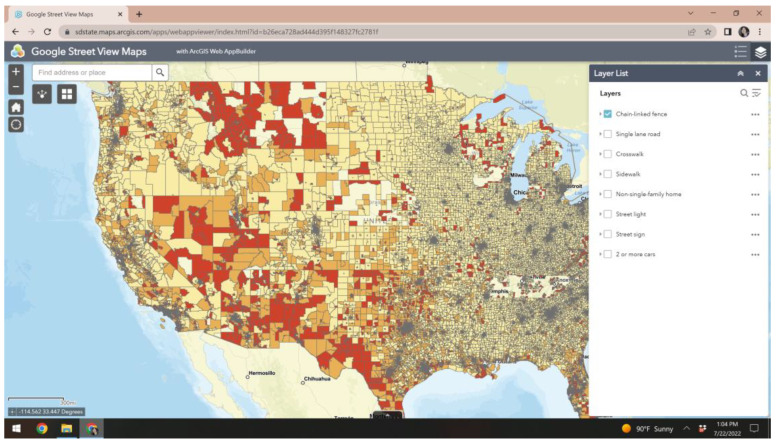
Geoportal interface. Darker colors signal higher prevalence of a neighborhood characteristic.

We constructed a publicly available geoportal to allow users to dynamically interact with the derived GSV neighborhood data (Figure 2). Users can navigate to the geoportal here: https://arcg.is/88nK40 (accessed on 2 September 2022). Users are able to:

- Select the GSV variable to display (e.g., sidewalk);
- Type a location or address in the search bar and the map will zoom to that area
- Darker colors signal higher prevalence of neighborhood feature

### 2.3. Demographic and Socioeconomic Data

Our analyses accounted for census tract population density, household size, median age, household income, poverty rate, unemployment, percent with less than a high school education, percent Asian, percent Black, and percent Hispanic. Covariate information was obtained from the American Community Survey 2018 5-year estimates, with the exception of population density and household size, which were obtained from the 2010 US Census.

### 2.4. Health Outcome Data

Census tract level health data came from the PLACES 2021 Release. PLACES, funded by the Robert Wood Johnson Foundation and the CDC Foundation, has extended the original 500 Cities project. The dataset includes measures of chronic disease risk, health outcomes, health status, and preventive services for local areas across the United States. 

Health outcomes data were derived from the 2018 and 2019 BRFSS self-reported data on chronic health conditions in which the respondents reported that a healthcare professional told them they had the following conditions: obesity (BMI ≥ 30), high blood pressure, high cholesterol, diabetes (other than diabetes during pregnancy), cancer (other than skin cancer), and depressive disorder. Poor mental health days were operationalized as a respondent reporting ≥ 14 days in the past 30 days during which their mental health was not good. Health behaviors examined included self-reported inadequate sleep (<7 h/night) and current smoking (smoked ≥ 100 cigarettes in their lifetime and currently smoke every day or some days). PLACES 2021 data are available at the county, place, zip code, and census tract levels. We chose the census tract level to approximate neighborhood boundaries. More information about the methodology can be found at www.cdc.gov/places (accessed on 2 September 2022).

### 2.5. Statistical Analyses

Descriptive statistics were estimated for built environment characteristics, sociodemographics, and health outcomes. National maps display the geographical distribution of built environment characteristics. We fit adjusted linear regression models to estimate associations between GSV-derived built environment characteristics and health outcomes, controlling for potential confounding variables such as racial/ethnic composition and economic disadvantage. Separate regressions were run for each built environment indicator given low to moderate associations between the built environment indicators that varied from −0.17 for single lane roads and sidewalks to 0.82 for street signs and two or more cars. Stata IC15 (StataCorp LP, College Station, TX, USA) was used for all statistical analyses. This study was approved by the University of Maryland Institutional Review Board.

## 3. Results

Table 1 displays descriptive statistics of the built environment characteristics, demographics, and health characteristics summarized at the census tract level in the United States. Single lane roads were very common in GSV images, with an average census tract level prevalence estimate of 67%. Additionally, on average, about 44% of GSV images at the census tract level had a sidewalk and 36% had two or more cars. About 30% had the presence of a non-single family home (e.g., apartment, commercial building). Dilapidated buildings had a prevalence of 24%, as did street signs. Street lights (16%) had a lower prevalence, and crosswalks were the rarest built environment feature at 3.6% (Table 1). Across census tracts in the United States, percent college educated was about 28% and percent Black and Hispanic was 14% and 15%, respectively. Chronic conditions such as obesity, high blood pressure and high cholesterol were very common with prevalence of 32–33% among adults. Diabetes occurred with a prevalence of 11% at the census tract level. Depression was high with a prevalence of 37%. Current smoking was common with a prevalence of 18%. About 18% of adults reported sleeping less than 7 h a night.

**Figure 3:**
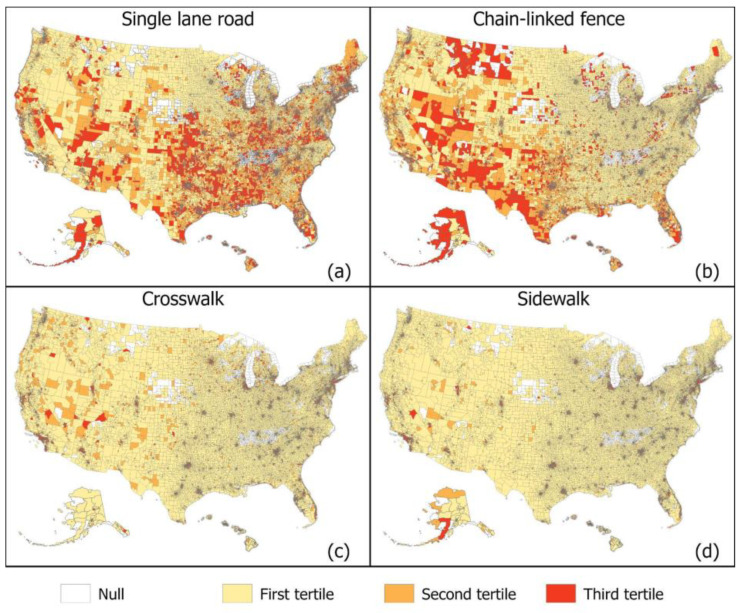
Census tract level distribution of GSV-derived neighborhood characteristics. (**a**) single lane roads, (**b**) chain-link fence, (**c**) crosswalk, (**d**) sidewalk. Darker colors signal higher prevalence of a neighborhood characteristic.

**Figure 4:**
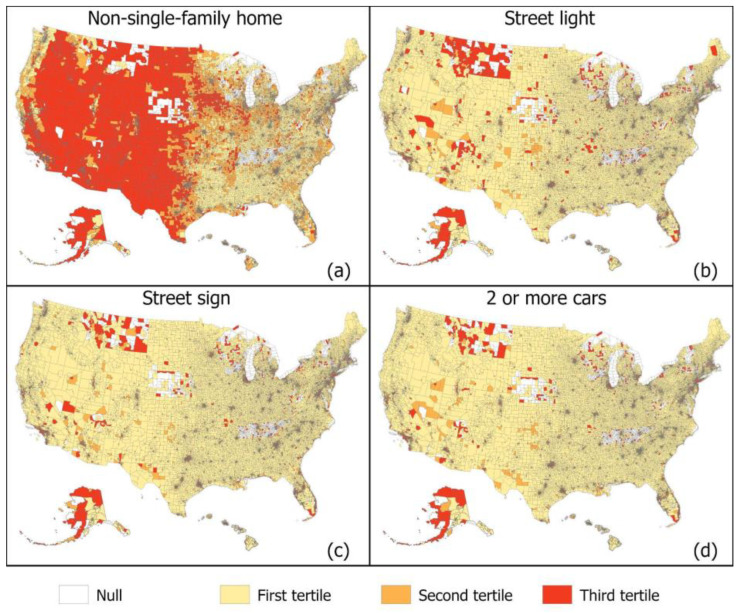
Census tract level distribution of GSV-derived neighborhood characteristics. (**a**) non-single family home, (**b**) street light, (**c**) street sign, (**d**) two or more cars. Darker colors signal higher prevalence of a neighborhood characteristic.

**Figure 5:**
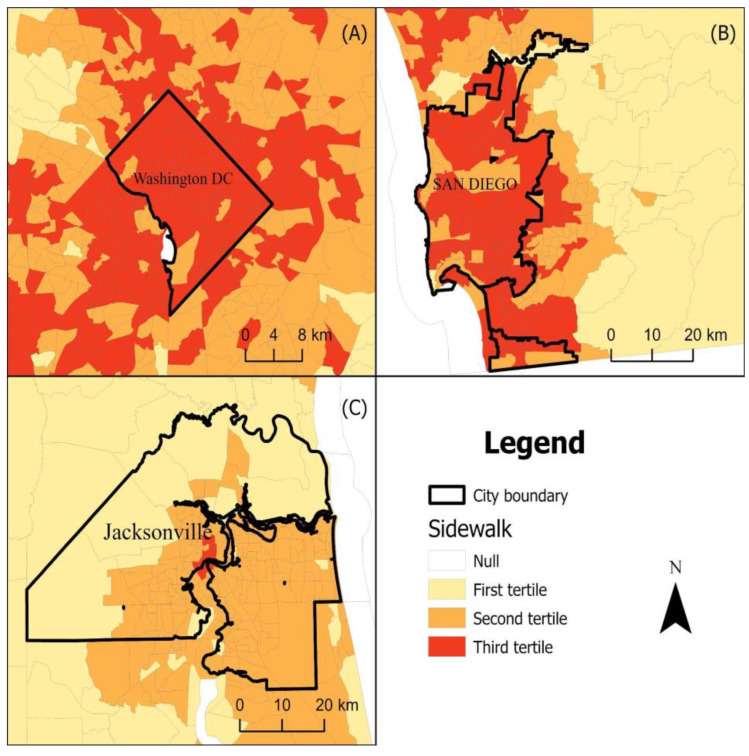
Geographic distribution of sidewalks across census tracts in three metropolitan areas. (**A**) Washington DC, (**B**) San Diego, California, (**C**) Jacksonville, Florida. Darker colors signal higher prevalence of a neighborhood characteristic.

Figure 3 and Figure 4 displays national maps of our GSV-derived built environment indicators geographical dispersion and variation which seem to follow different patterns for each indicator. For example, single lane roads seem to be dispersed more commonly in the south and northeast (Figure 3). Alternatively, neighborhoods composed of purely single-family homes appear more prominent eastern half of the United States (Figure 4). Figure 5 zooms into three metropolitan areas to show additional local variation that can get lost in national maps. From Figure 5, we see that sidewalks are most prevalent in Washington DC followed by San Diego and then Jacksonville Florida, which only has plentiful sidewalks in one area. From the maps, we see that sidewalks for these areas tend to concentrate in the centroid and become less prominent in outlying areas. Table 2 displays the regression results. The estimates from the crude models were generally stronger in magnitude than those from the adjusted models that accounted for differences across census tracts in sociodemographics. Nonetheless, even in the adjusted models, consistent associations were observed between built environments and a variety of important health outcomes. Single lane roads, an indicator of lower urban development, were linked with a higher prevalence of chronic conditions. For example, census tracts in the third tertile for single lane roads had an obesity prevalence that was 1.34% (95% CI 1.26, 1.42) higher than areas in the first tertile. The two or more cars indicator was used to characterize areas with more activity, and it was found to be related to lower chronic conditions. Census tracts in the third tertile for two or more cars had an obesity prevalence 3.39% (95% CI: −3.48, −3.30) lower than those in the first tertile. Street signs and street lights can help people navigate neighborhoods, and they were also found to be associated with a reduced burden of chronic conditions. Indicators of walkability such as sidewalks, crosswalks, and non-single family homes were related to lower chronic conditions. Living in a census tract in the third tertile for sidewalks is associated with about a 3.1% reduction in obesity and high blood pressure and a 1.9% reduction in high cholesterol.

Table 3 displays associations between built environment characteristics and mental health and health behaviors. Single lane roads (the indicator of lower urban development) were associated with more poor mental health days, depression, current smoking, and sleeping less than 7 h (only 3rd tertile). The chain-link fence (the indicator of physical disorder) was associated with higher depression and poor mental health days (only 3rd tertile). However, chain-link fences were unexpectedly associated with lower current smoking and lower inadequate sleep. However, the other indicators of urbanicity and walkability that were examined in this study were uniformly associated with better mental health and health behaviors. In particular, the 3rd tertile of crosswalks, sidewalks, and two or more cars were associated with a 1.7–2.0% reduction in current smoking. Additionally, crosswalks, sidewalks, and non-single family homes were associated with a 1.3–1.5% reduction in depression.

## 4. Discussion

Our analysis of 164 million GSV images examined the associations between built environment characteristics and neighborhood health, contributing a unique methodology to an expanding body of research exploring the potential impacts of place characteristics on health. We identified three key markers of urban development: the presence of two or more cars, street signs, and street lights. Our study found that these markers of urban development were associated with lower obesity, high blood pressure, high cholesterol, diabetes, as well as cancer. These markers were also linked with a lower prevalence of poor mental health days, depression, and current smoking. Crosswalks, sidewalks, and non-single family homes, which were used as measures of walkability, were associated with a lower prevalence of chronic conditions, depression, and current smoking. Single lane roads, which were utilized as an indication of lower levels of urban development, were linked with a higher burden of chronic conditions. Chain-link fences (indicator of physical disorder) were associated with higher depression and poor mental health days. However, chain-link fences were unexpectedly associated with lower current smoking and lower inadequate sleep. Moreover, although there are studies [37,38,39,40] that have established associations between neighborhood indicators of physical disorders and health, we have not found any that examine the influence of chain-link fences and health outcomes. Further investigation is warranted on this particular neighborhood indicator and its complex relationship with different domains of health.

Markers of urban development were robustly linked with lower chronic health conditions. This association has been corroborated by our past projects that recognize built characteristics of urbanization often increase physical activity, health care accessibility, and additional resources within a community, thereby contributing to improved health [41,42]. While non-single family homes were linked with reductions in all measured adverse health outcomes and risk behaviors, street signs and two or more cars were found to have positive associations with inadequate sleep (3rd tertile only). This may be attributed to increased noise and traffic that are often symptoms of urban development, resulting in an increase in measured risk behavior rather than a reduction consistent with other indicators of health [43]. 

Our findings underscore the large health disparity that exists between people living in rural and urban areas in the United States [44]. Residents of rural areas are at a higher risk of geographic isolation which contributes to limited access to healthy food sources [45], have fewer opportunities for physical activity [46], and are at a higher risk of smoking [47,48] compared to their urban counterparts. Rural areas’ geographic isolation also extends to inadequate access to health care and healthcare providers [49]. Although studies have not been able to establish a direct link between urbanicity and sleep deprivation [50], researchers argue that other negative health factors that disproportionately affect rural areas such as older age [51], limited access to health care, inadequate physical activity, longer commutes, and poor health, are consequential in sleep deficiency [52].

Our findings aligned with frequent associations between features that enhance walkability and a lower prevalence of chronic health conditions. Sidewalks were correlated with a profound reduction in levels of obesity and high blood pressure, with reductions in the 3rd tertile of −3.1% for both conditions. Previous studies using footprint-level data as a measure of neighborhood walkability corroborated our findings, independently associating increased walkability with reduced risks of hypertension [53]. In our models, neighborhood walkability was also correlated with reduced depression and poor mental health days, a relationship likely stemming from multiple factors. Built features of walkability have been found to promote physical activity through overall increases in transportational walking [54]; in addition, sidewalk availability has been independently associated with an increased step count in a study that evaluated four other indicators of walkability: population density, street connectivity, and access to transportation and destinations [55]. There is mounting evidence of the relationship between increased physical activity and improved mental health, including decreased levels of suicidal ideation [56], and decreased symptoms of depression and anxiety [57]. Moreover, previous literature has linked increased walkability with decreased depressive symptoms in meneven when adjusted for physical activity [58], which can partially be attributed to the fact that walkability can reduce loneliness, especially in elderly populations [59]. 

In addition, our study found that crosswalks exhibited the strongest associations with diabetes and high cholesterol than any other measured characteristic, with reductions of −1.9% and −3.1%, respectively. Recent studies leveraging GSV images have linked crosswalks with reduced premature mortality and physical inactivity [60,61]. Sidewalks had a positive association with inadequate sleep. We used sidewalks as an indicator for walkability: more sidewalks allow more pedestrians. Therefore, some possible explanations could be related to higher neighborhood noise level and lower safety, which both have significant associations with negative sleep outcomes [62]. However, in our result, crosswalks showed a negative association with inadequate sleep. This could be caused by how crosswalks and sidewalks are used differently in city design. In general, crosswalks appear in larger open areas, used at road intersections, whereas sidewalks seem to be closer to residential buildings that any negative outcomes from sidewalks could affect residents more distinctly. Another study also provides evidence on how crosswalks can improve health outcomes [61]. Further research examining sidewalks’ and crosswalks’ different functionalities could help clarify the mechanisms by which they influence health. Despite these notable benefits to health, crosswalks were the rarest built environment feature, with an average prevalence of only 3.6% at the census tract level. It is worth exploring further the benefits that crosswalks have on adult health outcomes. Crosswalks may be an underutilized lever for improving population health.

### Study Strengths and Limitations

Using GSV images allows for national assessment of neighborhood conditions, which would not be possible with other methods such as on-site neighborhood audits or even virtual audits with manual annotations. This study utilized over 164 million images across the land mass of the United States. The national scale enables us to examine empirically whether certain neighborhood features are associated with important drivers of morbidity and mortality in the United States. 

Although we were able to establish compelling associations between built environment indicators and health outcomes, our study has certain limitations. Data collection for the study occurred in November 2019 when we downloaded the most recent image available for each sampling point through the Google Street View API. However, areas across the United States differ with regard to the frequency of their GSV image updates. As such, images in our dataset have dates ranging from 2007 to 2019 with the median year of 2015. The time band means that the image data might not accurately capture certain neighborhood conditions for certain areas. This is particularly common in rural areas for which GSV data are not as frequently updated as in urban areas, possibly resulting in a differential measurement bias. Moreover, utilizing computer vision technology came with certain limitations. For instance, the use of supervised learning models narrowed the possible neighborhood characteristics to those that can be labeled with good reliability between annotators, specifically neighborhood characteristics that are generally not too small visually and not too subjective. Computer vision algorithms struggle with small objects like litter, rare characteristics like graffiti, and features with variable appearance such as dilapidated buildings. Subjective characteristics complicate prediction. Ratings for road and building condition varied substantially across reviewers depending on their viewpoints regarding what constitutes dilapidation. Crowdsourcing techniques that draw on resident and visitor ratings might offer a means of overcoming the subjective classification challenge by establishing area-level ratings that reveal the variability and stability in subjective perceptions of neighborhoods. No single dataset can capture all relevant features of a given community. Among the characteristics that image data cannot ascertain are neighborhood residents’ perceptions including whether they feel safe walking in their community. Moreover, unlike on-site visits, annotations of GSV images did not provide the same depth of understanding of context. For example, on-site visits allow the annotator to observe the noise of a place, variation or homogeneity of adjacent spaces, business patterns, traffic and pedestrian flows, and residents’ interactions and perceptions. The use of complementary datasets such neighborhood surveys and administrative data can further enrich GSV annotations. 

## 5. Conclusions

The difficulties of characterizing built environments at large geographical scales hinder the possibilities of exploring connections between neighborhood characteristics and health outcomes on a national level. To enrich studies in this field and help provide insights for public health optimization, we utilized 164 million GSV images and leveraged computer vision models. This study highlights the utility of virtual audits for characterizing neighborhood features that have important implications for physical diseases, mental health and behavioral issues. Our study results suggest that indicators such as neighborhood walkability (crosswalks, sidewalks, and non-single-family homes) and urban development (two or more cars, street signs, and streetlights) are connected with lower chronic disease, better mental health, and reduced smoking. As an indicator of lower levels of urban development, single-lane roads were associated with higher levels of chronic disease. Moreover, as a physical disorder indicator, chain link fences were linked with poor mental health. We see the importance of neighborhood walkability in building up our bodies and decreasing the chance of getting depression and anxiety by increasing social contacts which are consistent with our results. As such, altering the built environment might be an effective lever for reducing adverse health outcomes and improving population health. Additionally, leveraging computer vision technology and relatively new data sources are enabling larger studies on the impacts of the built environment on health. Continuing to develop these technologies can pave the way for additional advances and make neighborhood studies more cost-efficient, timely, and sustainable. The theoretical contributions of this study include its highlight of features of the neighborhood built environment with implications for population health which may help to guide future research. Often, public health research is driven by the search for individual-level factors such as behaviors or treatments such as drug therapy. However, our study points to the contributions of environmental factors for shaping health. The practical contribution of this study includes the identification of other potential levers to change population health. For instance, health organizations can team up with city planners to structure neighborhood environments that are conducive to health by emphasizing features that would increase walkability and access to recreational and other neighborhood resources. This study also calls for additional studies on built environment indicators; limited studies are available explaining how certain built environment characteristics promote health or are detrimental to health, so continued explorations are necessary.

## Figures and Tables

**Table 1:** Descriptive statistics of neighborhood characteristics and health outcomes, census tract.

	N	Mean (SD)
*Built environment characteristics*		
Crosswalks	70,359	3.63 (4.37)
Sidewalks	70,359	43.96 (30.72)
Single lane road	70,359	67.11 (14.57)
Presence of apartment/commercial building	70,359	29.80 (23.69)
Streetlights	70,319	15.66 (14.96)
Street signs	70,344	24.28 (15.08)
2 or more cars	70,288	36.10 (20.53)
Chain Link fence	70,311	7.63 (13.79)
*Census tract characteristics*		
Population size	72,864	4237.29 (1972.52)
Percent 65 years+	72,578	13.63 (7.39)
Percent male	72,578	49.18 (4.05)
Percent Black	72,578	13.83 (22.29)
Percent Hispanic	72,578	15.27 (20.82)
Percent single female headed households	72,472	13.65 (8.17)
Percent owner-occupied housing	72,472	64.32 (22.50)
Percent college educated	72,436	27.67 (18.50)
Median household income	72,048	67,432.68 (32,960.44)
Percent unemployed	72,330	10.36 (6.34)
Child opportunity index, range 0 to 100	72,213	49.15 (28.61)
*Adult health outcomes*		
Obesity	70,338	32.63 (6.82)
Diabetes	70,338	10.96 (3.73)
High Blood Pressure	70,338	32.49 (7.36)
High Cholesterol	70,338	31.83 (4.79)
Cancer	70,338	6.73 (1.94)
Poor mental health days	70,338	15.21 (3.57)
Depression	72,337	36.77 (5.23)
Sleep less than 7 h a night	70,338	17.61 (3.45)
Current Smoking	70,338	17.98 (5.76)

This table presents summary statistics, mean and standard deviation (SD) for variables included in the analysis. Data were aggregated to the census tract level. N = number of census tracts. Built environment characteristics were derived from Google Street View images. Sociodemographic characteristics came from the American Community Survey 2018 5-year estimates and the 2010 US Census. Health outcomes data came from PLACES 2021.

**Table 2:** Built environment predictors of adult health outcomes ^a^.

	Obese	High Blood Pressure	High Cholesterol	Diabetes	Cancer
*Built Environment Characteristics*	Crude Odds Ratio (95% CI)	Crude Odds Ratio (95% CI)	Crude Odds Ratio (95% CI)	Crude Odds Ratio (95% CI)	Crude Odds Ratio (95% CI)
Single lane road					
3rd tertile (highest)	2.19 (2.06, 2.31)	3.23 (3.09, 3.36)	2.18 (2.09, 2.26)	0.75 (0.69, 0.82)	0.69 (0.66, 0.73)
2nd tertile	1.11 (0.99, 1.24)	1.71 (1.58, 1.84)	1.41 (1.33, 1.50)	0.21 (0.14, 0.28)	0.50 (0.46, 0.53)
2 or more cars					
3rd tertile (highest)	−1.97 (−2.09, −1.84)	−3.46 (−3.60, −3.33)	−4.43 (−4.51, −4.34)	0.34 (0.27, 0.40)	−1.80 (−1.84, −1.77)
2nd tertile	−1.46 (−1.58, −1.33)	−2.15 (−2.29, −2.02)	−2.20 (−2.28, −2.12)	−0.33 (−0.40, −0.26)	−0.71 (−0.75, −0.68)
Street signs					
3rd tertile (highest)	−2.44 (−2.56, −2.31)	−4.45 (−4.58, −4.32)	−4.68 (−4.76, −4.60)	−0.05 (−0.12, 0.02)	−1.96 (−2.00, −1.93)
2nd tertile	−1.02 (−1.14, −0.89)	−2.04 (−2.17, −1.91)	−2.55 (−2.63, −2.47)	−0.27 (−0.34, −0.20)	−0.81 (−0.84, −0.77)
Street lights					
3rd tertile (highest)	−1.49 (−1.62, −1.37)	−3.04 (−3.17, −2.91)	−3.89 (−3.97, −3.81)	0.35 (0.28, 0.42)	−1.64 (−1.68, −1.61)
2nd tertile	−0.86 (−0.99, −0.74)	−2.74 (−2.87, −2.60)	−2.82 (−2.91, −2.74)	−0.54 (−0.61, −0.47)	−0.89 (−0.92, −0.86)
Non-single family home					
3rd tertile (highest)	−1.58 (−1.70, −1.45)	−3.56 (−3.70, −3.43)	−3.77 (−3.85, −3.69)	0.12 (0.05, 0.19)	−1.60 (−1.63, −1.56)
2nd tertile	−0.08 (−0.21, 0.04)	−1.20 (−1.33, −1.07)	−1.43 (−1.51, −1.34)	0.06 (−0.01, 0.13)	−0.59 (−0.62, −0.55)
Sidewalks					
3rd tertile (highest)	−4.09 (−4.21, −3.96)	−5.83 (−5.95, −5.70)	−5.06 (−5.13, −4.98)	−0.94 (−1.01, −0.87)	−1.82 (−1.85, −1.79)
2nd tertile	−2.33 (−2.45, −2.21)	−3.23 (−3.36, −3.10)	−2.85 (−2.92, −2.77)	−0.78 (−0.85, −0.71)	−0.77 (−0.81, −0.74)
Crosswalks					
3rd tertile (highest)	−4.49 (−4.61, −4.37)	−5.99 (−6.12, −5.86)	−4.86 (−4.94, −4.78)	−1.25 (−1.32, −1.18)	−1.57 (−1.61, −1.54)
2nd tertile	−1.84 (−1.96, −1.72)	−2.68 (−2.81, −2.55)	−2.40 (−2.48, −2.32)	−0.58 (−0.65, −0.51)	−0.68 (−0.71, −0.65)
N	67,445	67,445	67,445	67,445	67,445
	**Adjusted Odds Ratio (95% CI) ^b^**	**Adjusted Odds Ratio (95% CI) ^b^**	**Adjusted Odds Ratio (95% CI) ^b^**	**Adjusted Odds Ratio (95% CI) ^b^**	**Adjusted Odds Ratio (95% CI) ^b^**
Single lane road					
3rd tertile (highest)	1.34 (1.26, 1.42)	1.15 (1.08, 1.21)	0.65 (0.60, 0.70)	0.34 (0.31, 0.38)	0.11 (0.10, 0.12)
2nd tertile	0.76 (0.68, 0.83)	0.67 (0.60, 0.73)	0.35 (0.30, 0.40)	0.14 (0.11, 0.18)	0.08 (0.07, 0.09)
2 or more cars					
3rd tertile (highest)	−3.39 (−3.48, −3.30)	−2.90 (−2.98, −2.82)	−1.67 (−1.74, −1.61)	−1.23 (−1.28, −1.19)	−0.37 (−0.38, −0.36)
2nd tertile	−0.98 (−1.06, −0.90)	−1.55 (−1.61, −1.48)	−1.05 (−1.10, −0.99)	−0.72 (−0.76, −0.69)	−0.18 (−0.19, −0.17)
Street signs					
3rd tertile (highest)	−2.71 (−2.81, −2.62)	−2.34 (−2.42, −2.26)	−1.32 (−1.39, −1.26)	−0.92 (−0.97, −0.88)	−0.31 (−0.32, −0.30)
2nd tertile	−1.11 (−1.19, −1.03)	−1.44 (−1.51, −1.38)	−0.87 (−0.92, −0.81)	−0.68 (−0.72, −0.65)	−0.15 (−0.16, −0.14)
Street lights					
3rd tertile (highest)	−1.56 (−1.65, −1.48)	−0.83 (−0.87, −0.80)	−1.99 (−2.07, −1.92)	−1.36 (−1.42, −1.30)	−0.28 (−0.29, −0.27)
2nd tertile	−0.69 (−0.77, −0.61)	−0.62 (−0.65, −0.58)	−1.37 (−1.44, −1.30)	−1.00 (−1.05, −0.94)	−0.15 (−0.16, −0.14)
Non-single family home					
3rd tertile (highest)	−1.90 (−1.99, −1.81)	−1.59 (−1.67, −1.52)	−1.00 (−1.06, −0.94)	−0.60 (−0.64, −0.56)	−0.19 (−0.20, −0.18)
2nd tertile	−0.38 (−0.46, −0.31)	−0.67 (−0.74, −0.61)	−0.45 (−0.50, −0.40)	−0.27 (−0.30, −0.23)	−0.09 (−0.10, −0.08)
Sidewalks					
3rd tertile (highest)	−3.07 (−3.16, −2.97)	−3.12 (−3.20, −3.04)	−1.85 (−1.91, −1.79)	−1.13 (−1.17, −1.09)	−0.34 (−0.35, −0.32)
2nd tertile	−1.07 (−1.15, −0.98)	−1.71 (−1.78, −1.64)	−1.20 (−1.25, −1.14)	−0.75 (−0.79, −0.71)	−0.14 (−0.15, −0.13)
Crosswalks					
3rd tertile (highest)	−2.99 (−3.08, −2.90)	−1.29 (−1.33, −1.25)	−3.07 (−3.14, −2.99)	−1.85 (−1.91, −1.79)	−0.28 (−0.29, −0.27)
2nd tertile	−0.80 (−0.88, −0.72)	−0.63 (−0.67, −0.60)	−1.46 (−1.52, −1.39)	−0.96 (−1.01, −0.90)	−0.10 (−0.11, −0.09)
N	67,167	67,167	67,167	67,167	67,167

^a^ Data source for health outcome: CDC PLACES 2021. ^b^ Adjusted Linear regression models were run for each outcome separately. Models controlled for census tract population size, percent of the population 65 years and older, percent male, percent Hispanic, percent black, median household income, percent female headed households, and percent owner occupied housing, percent with a college degree, percent employed, and child opportunity index. Built environment characteristics were categorized into tertiles, with the lowest tertile serving as the referent group. Standard errors adjusted for clustering of values within a census tract.

**Table 3:** Built environment predictors of adult mental health and risk behaviors ^a^.

	Poor Mental Health Days	Depression	Inadequate Sleep (<7 h a Night)	Current Smoking
*Built Environment Characteristics*	Adjusted Odds Ratio (95% CI) ^b^	Adjusted Odds Ratio (95% CI) ^b^	Adjusted Odds Ratio (95% CI) ^b^	Adjusted Odds Ratio (95% CI) ^b^
Single lane road				
3rd tertile (highest)	0.51 (0.48, 0.55)	0.82 (0.78, 0.87)	0.19 (0.13, 0.24)	0.82 (0.76, 0.87)
2nd tertile	0.32 (0.28, 0.35)	0.60 (0.55, 0.64)	−0.19 (−0.25, −0.14)	0.35 (0.30, 0.41)
Chain-linked fence				
3rd tertile (highest)	0.17 (0.12, 0.21)	0.43 (0.37, 0.48)	−0.30 (−0.37, −0.24)	−0.58 (−0.65, −0.52)
2nd tertile	−0.14 (−0.17, −0.10)	0.10 (0.05, 0.14)	−0.40 (−0.45, −0.35)	−0.80 (−0.85, −0.75)
Crosswalks				
3rd tertile (highest)	−0.80 (−0.84, −0.76)	−1.29 (−1.35, −1.23)	−0.56 (−0.62, −0.49)	−2.04 (−2.10, −1.97)
2nd tertile	−0.16 (−0.19, −0.12)	−0.35 (−0.40, −0.30)	−0.15 (−0.21, −0.09)	−0.68 (−0.74, −0.63)
Sidewalks				
3rd tertile (highest)	−0.89 (−0.93, −0.85)	−1.46 (−1.52, −1.40)	0.51 (0.44, 0.57)	−1.68 (−1.74, −1.61)
2nd tertile	−0.19 (−0.23, −0.16)	−0.37 (−0.42, −0.32)	0.01 (−0.05, 0.07)	−0.65 (−0.71, −0.60)
Non-single family home				
3rd tertile (highest)	−0.68 (−0.72, −0.64)	−1.37 (−1.43, −1.32)	−0.67 (−0.73, −0.60)	−1.11 (−1.17, −1.04)
2nd tertile	−0.31 (−0.35, −0.28)	−0.44 (−0.48, −0.39)	−0.82 (−0.88, −0.77)	−0.51 (−0.56, −0.45)
Street lights				
3rd tertile (highest)	−0.28 (−0.32, −0.25)	−0.80 (−0.86, −0.75)	−0.01 (−0.07, 0.05)	−1.02 (−1.09, −0.96)
2nd tertile	−0.18 (−0.21, −0.14)	−0.25 (−0.30, −0.20)	−0.11 (−0.16, −0.05)	−0.57 (−0.63, −0.52)
Street signs				
3rd tertile (highest)	−0.42 (−0.46, −0.38)	−0.81 (−0.87, −0.75)	0.57 (0.50, 0.64)	−1.23 (−1.30, −1.16)
2nd tertile	0.18 (−0.22, −0.15)	−0.30 (−0.35, −0.25)	−0.02 (−0.07, 0.04)	−0.72 (−0.77, −0.66)
2 or more cars				
3rd tertile (highest)	−0.67 (−0.72, −0.63)	−1.18 (−1.24, −1.12)	0.17 (0.10, 0.24)	−1.69 (−1.75, −1.62)
2nd tertile	−0.17 (−0.20, −0.13)	−0.34 (−0.39, −0.29)	0.04 (−0.02, 0.09)	−0.64 (−0.69, −0.58)
N	67,167	67,167	67,167	67,167

^a^ Data source for health outcome: CDC PLACES 2021. ^b^ Adjusted Linear regression models were run for each outcome separately. Models controlled for census tract population size, percent of the population 65 years and older, percent male, percent Hispanic, percent black, median household income, percent female headed households, and percent owner occupied housing, percent with a college degree, percent employed, and child opportunity index. Built environment characteristics were categorized into tertiles, with the lowest tertile serving as the referent group. Standard errors adjusted for clustering of values within a census tract.

## Data Availability

PLACES 2021 and the American Community Survey are publicly available. Google Street View neighborhood-level data can be accessed in the geoportal: https://arcg.is/88nK40 (accessed on 2 September 2022).
